# Rapid detection of influenza A viruses using a real-time reverse transcription recombinase-aided amplification assay

**DOI:** 10.3389/fcimb.2022.1071288

**Published:** 2023-01-05

**Authors:** Huan Cui, Cheng Zhang, Fei Tu, Kui Zhao, Yunyi Kong, Jie Pu, Lei Zhang, Zhaoliang Chen, Yuanyuan Sun, Yujie Wei, Chuncai Liang, Juxiang Liu, Jun Liu, Zhendong Guo

**Affiliations:** ^1^ Changchun Veterinary Research Institute, Chinese Academy of Agriculture Sciences, Changchun, China; ^2^ College of Animal Medicine, Jilin University, Changchun, China; ^3^ College of Veterinary Medicine, Hebei Agricultural University, Baoding, China

**Keywords:** reverse transcription recombinase-aided amplification (RT-RAA), influenza A viruses (IAVs), isothermal amplification, rapid diagnosis, clinical diagnosis

## Abstract

**Introduction:**

Influenza A viruses (IAVs) are important pathogens of respiratory infections, causing not only seasonal influenza but also influenza pandemics and posing a global threat to public health. IAVs infection spreads rapidly, widely, and across species, causing huge losses, especially zoonotic IAVs infections that are more harmful. Fast and sensitive detection of IAVs is critical for controlling the spread of this disease.

**Methods:**

Here, a real-time reverse transcription recombinase-aided amplification (real-time RT-RAA) assay targeting conserved positions in the matrix protein gene (M gene) of IAVs, is successfully established to detect IAVs. The assay can be completed within 20 min at 42°C.

**Results:**

The sensitivity of the real-time RT-RAA assay was 142 copies per reaction at 95% probability, which was comparable to the sensitivity of the RT-qPCR assay. The specificity assay showed that the real-time RT-RAA assay was specific to IAVs, and there was no cross-reactivity with other important viruses. In addition, 100%concordance between the real-time RT-RAA and RT-qPCR assays was achieved after testing 120 clinical specimens.

**Discussion:**

The results suggested that the real-time RT-RAA assay we developed was a specific, sensitive and reliable diagnostic tool for the rapid detection of IAVs.

## 1 Introduction

Influenza A viruses (IAVs) are highly contagious respiratory pathogens, belonging to the Orthomyxoviridae family of segmented and negative sense single-stranded RNA viruses that easily lead to seasonal influenza outbreaks and pose a threat to global public health (Nypaver et al., 2021; Zhang et al., 2021a). IAVs are divided into many subtypes according to different H (H1-H18) and N (N1-N11) antigens (Tong et al., 2013). The genome of IAVs is approximately 13.5 kb and encodes 11 proteins: HA, NA, NP, M1, M2, NS1, NS2, PA, PB1, PB2 and PB1-F2 (Ng et al., 2009; Chauhan and Gordon, 2020; Hao et al., 2020). IAVs have long endangered the global livestock industry and human health, and have attracted great attention (Zhang et al., 2021b). The development of a rapid, sensitive, efficient and accurate detection method for IAVs is helpful for the early clinical diagnosis, treatment, and control of the spread of influenza A. RT-qPCR assay is a sensitive tool to detect IAVs (Yang et al., 2022). However, complex operations and expensive equipment requirements limit its application in resource-limited environments and point-of-care testing (POCT). Thus, it is necessary to establish a fast, simple, low-cost, and sensitive on-site diagnostic method to detect IAVs.

Recombinase-aided amplification (RAA), a novel isothermal *in vitro* nucleic acid amplification technique, can be completed within 30 minutes at 37–42°C. There are three key enzymes in the system: a single-stranded DNA-binding protein (SSB), which is a protein specifically responsible for binding to single-stranded regions of DNA; recombinase (match the specific primers with template DNA); and strand-displacing DNA polymerase (for extension and DNA amplification) (Li et al., 2020; Tu et al., 2022). In the RAA system, adding specific fluorescent probes can monitor DNA amplicons in real-time. Combining reverse transcriptase with specific fluorescent probes can realize real-time monitoring of RNA amplicons (Tu et al., 2021; Wu et al., 2021; Tu et al., 2022). In addition, combining the CRISPR system with RAA technology enables ultrasensitive detection of DNA or RNA single molecules (Zhao et al., 2021; Duan et al., 2022; Zhang et al., 2022). Due to the characteristics of high sensitivity and specificity, low reaction time, simple operation, visual judgment of results, and suitability for on-site rapid diagnosis, RAA has been widely applied in the detection of various pathogenic microorganisms (Tu et al., 2021; Jiao et al., 2022; Wu et al., 2022).

Here, we designed the real-time RT-RAA primers and probes according to the conserved position of the IAVs *M* gene sequence, and successfully developed the real-time RT-RAA detection method for IAVs. The assay can be completed within 20 min. It is highly sensitive, specific, and easy to operate. Furthermore, we compared the detection performance of real-time RT-RAA and RT-qPCR methods using clinical samples.

## 2 Material and methods

### 2.1 Virus and clinical samples

IAVs (A/Sichuan/SC99/2019 (H1N1); A/Hebei/BD79/2018 (H3N2); A/chicken/Hebei/HB777/2006 (H5N1); A/chicken/Hebei/CK05/2019(H5N6); A/quail/Hebei/CH06-07/2018(H7N9); A/chicken/Hebei/015/2019(H9N2); A/environment/Fujian/EV01/2020(H11N3)), influenza B viruses (Victoria and Yamagata), influenza C virus, respiratory syncytial virus (A and B), SARS-CoV-2 BetaCoV/Beijing/IME-BJ05-2020 (Biological Sample Library: SAMC138020) and sixty-five clinical samples (lung tissue material, cloacal swab, oropharyngeal swab) were stored in the biosafety level 3 laboratory of Changchun Veterinary Research Institute, Chinese Academy of Agricultural Sciences. All samples were processed in a biosafety level 3 laboratory.

### 2.2 Design for primers and probe of real-time RT-RAA assay

**Figure 1:**
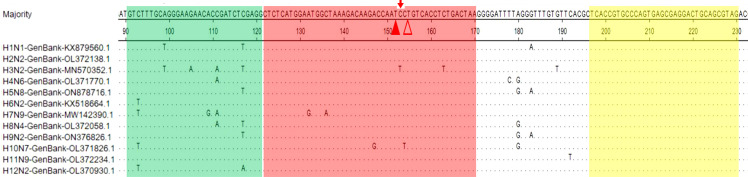
Locations of the real-time RT-RAA primers and probe on the *M* gene sequence of different IAVs strains. Use dots to represent nucleotide residues that match the majority. The forward primer (F91-121) is shaded in green; the reverse primer (R197-230) is blue and the exo probe (p122-170) is red. The two T residues within p122-170 labeled with a fluorophore (FAM) and quencher (BHQ1) are marked with solid and hollow triangles, respectively. THF is marked with arrow.

Twelve different strains of IAVs *M* gene sequences were aligned using DNASTAR software. SnapGene software (Version 4.3.6) was used to design primers and probes. According to the previously reported method to screen the optimal primers and probe (Tu et al., 2022). Furthermore, synthesizing the primers (M-F/M-R) and probe (M-P) of the M-based RT-qPCR assay for IAVs according to the previously reported (Yang et al., 2022)(**Table 1**). Details of the final primers and probes for real-time RT-RAA detection we designed in this study are shown in **Figure 1** and **Table 1**. Primers and probes were synthesized by Comate Biotech Co., Ltd (Changchun, China).

**Table 1:** The primers and probes used in IAVs real-time RT-RAA and RT-qPCR assays.

Name	Sequences (5’→3’)	Gene	Product	Source
F91-121	GTCTTTGCTGGGAAGAACACCGATCTTGAGG	*M*	140 bp	This study
R197-230	CTACGCTGCAGTCCTCGCTCACTGGGCACGGTGA	*M*		This study
p122-170 ^a^	CTCTCATGGAATGGCTAAAGACAAGACCAA(FAM-dT) (THF)C(BHQ1-dT)GTCACCTCTGACTAA[C3-spacer]	*M*		This study
M-F	CTTCTAACCGAGGTCGAA ACG	*M*	165 bp	Yang et al, 2022
M-R	CTTTAGCCACTCCATGAGAGC	*M*		Yang et al, 2022
M-P ^b^	FAM-CCTCAAAGCCGAGATC-MGB	*M*		Yang et al, 2022

a FAM-dT, thymidine nucleotide carrying fluorescein; BHQ1-dT, thymidine nucleotide carrying black hole quencher 1; THF: tetrahydrofuran spacer; C3-Spacer, C3 spacer at the 3’ end to block elongation.

b FAM, 6-carboxyfluorescein; MGB, minor groove binder.

### 2.3 Real-time RT-RAA assay

The assays were performed using a kit (#WLRE8208KIT) from Amp-Future Biotech Co., Ltd (China). Briefly, real-time RT-RAA system (25 μL per reaction): 14.7 μL Buffer A, 1.0 μL forward primer (10 μM), 1.0 μL reverse primer (10 μM), 0.3 μL exo probe (10 μM), 4.75 μL nuclease-free water, 2.0 μL nucleic acid template and 1.25 μL Buffer B (Note, buffer B is added last to the tube lids, close the tube lids carefully, vortex shortly and centrifuge briefly). Then the reaction tubes were placed into the 7500 Real-Time PCR System (Applied Biosystems) with a setting of 1 cycle per min for 20 min at 42°C. Real-time monitoring of the fluorescent signals. The highest conversion rate could be obtained using the reverse transcriptase at a temperature of 42°C according to the reagent instructions and our previous research (Tu et al., 2021).

### 2.4 RT-qPCR assay

The RT-qPCR reaction system of IAVs is described as follows: a 25 μL reaction mixture comprising 12.5 μL 2×One Step PrimeScript III RT-qPCR Mix, 9 μL nuclease-free water, 0.5 μL each of forward primer, reverse primer and probe (10 μM), and 2.0 μL nucleic acid template. The reaction was performed in a 7500 Real-Time PCR System (Applied Biosystems) with a setting of an initial step at 52°C for 5 min, then 95°C for 10 s, followed by 40 cycles of amplification (95°C for 5 s, 60°C for 30 s).

### 2.5 Analytical specificity

Evaluating the specificity of the real-time RT-RAA assay for IAVs detection using IAVs (H1N1, H3N2, H5N1, H5N6, H7N9, H9N2, and H11N3), IBV-V, IBV-Y, ICV, RSV-A, RSV-B, and SARS-CoV-2 pathogens.

### 2.6 Analytical sensitivity

After diluting the IAVs-M plasmid (pMD18-T-M) in a 10-fold ratio, DNA concentrations ranging from 1×10^5^ to 1×10^0^ copies per 2 μL were obtained. Two microliters of each dilution were used as a template to assess the real-time RT-RAA sensitivity. For comparison, the same template was tested in parallel using the RT-qPCR assay for IAVs. For a more accurate analysis of the limit of detection, eight independent runs were performed in both assays using the dilution series (10^5^–10^0^ copies per reaction) as templates, and probit regression analysis was performed on the data using IBM’s Statistical Product and Service Solutions (SPSS) software. The DNA copy number was calculated using the following formula: DNA copy number/μL = [plasmid concentration (ng/μL) × 10^-9^ × 6.02× 10^23^]/[DNA length (nt) × 660].

### 2.7 Detection of clinical samples

One hundred and twenty clinical samples (lung tissue material, cloacal swab, oropharyngeal swab) were tested by the real-time RT-RAA assay. The details of the clinical samples are provided in **Supplementary Table 1**. For comparison, the same samples were tested in parallel using the RT-qPCR assay for IAVs. Viral RNAs and total RNA from each sample were extracted using TRIzol reagent (Magen, Guangzhou, China) following the manufacturer’s instructions. The extracted RNA was eluted into 50 μL of nuclease-free water and stored at −80°C until needed.

### 2.8 Statistical analysis

The probit regression analysis was performed at the 95% probability level to determine the detection limits. The kappa and p values of real-time RT-RAA and RT-qPCR assays were calculated. Data analysis using IBM’s SPSS software.

## 3 Results

### 3.1 Locations of amplicon targets on *M* gene in IAVs genome

The *M* gene sequences of twelve different IAVs strains were aligned using DNASTAR software. SnapGene software was used to design specific primers and probe in conserved regions of the *M* gene. The two modified thymine (T) residues in our chosen probe (p122-170) are fully conserved among the twelve representative IAVs strains (**Figure 1**). Use dots to represent nucleotide residues that match the majority. After secondary primer screening, the optimal primer pair F91-121/R197-230 was screened out. The forward primer (F91-121) is shaded in green; the reverse primer (R197-230) is yellow and the exo probe (p122-170) is red. The two T residues within p122-170 labeled with a fluorophore (FAM) and quencher (BHQ1) are marked as solid and hollow triangles, respectively (**Figure 1**).

### 3.2 Screening primers

**Figure 2:**
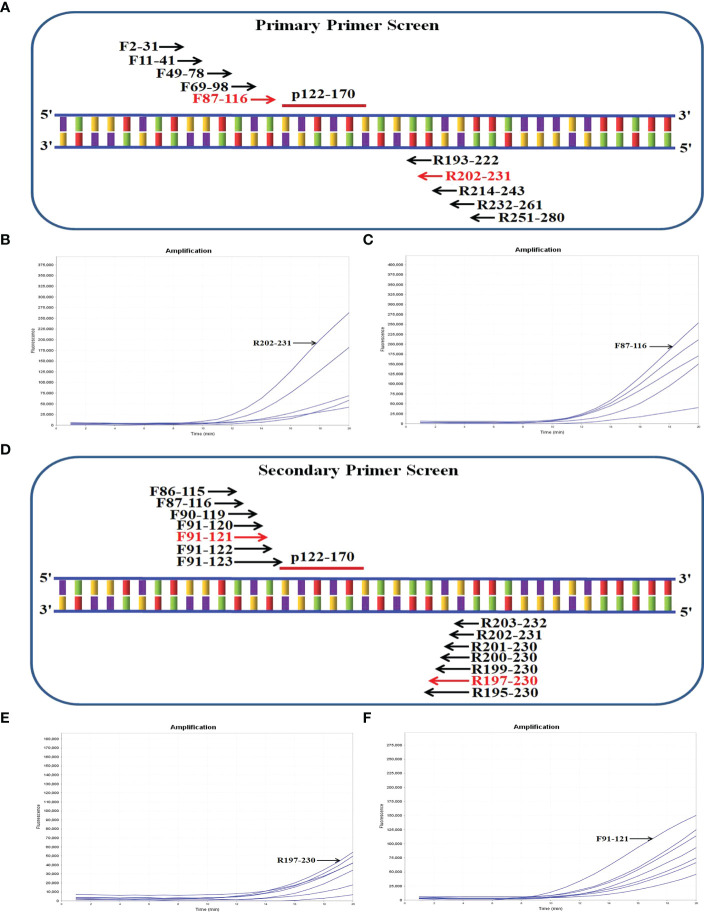
Screening the optical primers for real-time RT-RAA detection. **(A)** Sketch map of primary primer screening. In the primer name, the numbers indicate the position within the *M* gene from H1N1 (GenBank accession no. KX879560.1). **(B)** Primary reverse primer screening results. The forward primer F2-31 was randomly selected to screen all five reverse primers. **(C)** Primary forward primer screening results. The picked reverse primer R202-231 was used to pick all five forward primers. **(D)** Sketch map of secondary primer screening. **(E)** Secondary reverse primer screening results. The picked forward primer F87-116 was applied to screen all seven reverse primers. **(F)** Secondary forward primer screening results. The picked reverse primer R197-230 was used to pick all seven forward primers.

First, we selected an ideal probe (p122-170) (**Figure 2**; **Table 1**). Then, we designed five forward (F2-31, F11-41, F49-78, F69-98, F87-116) and five reverse (R193-222, R202-231, R214-243, R232-261, R251-280) candidate primers surrounding p122-170 (**Figure 2A**). The primer screening strategy was reported in previous studies (Tu et al., 2022). Briefly, we used the forward primer F2-31 (we randomly selected) to screen all five reverse primers, and the reverse primer R202-231 showed the best amplification (**Figure 2B**). Then R202-231 was used to screen all five forward primers, and F87-116 exhibited the best amplification (**Figure 2C**). Thus, the primer pair F87-116/R202-231 was the best in the primary primer screen. To screen for better primer pairs, we designed six new forward primers and six new reverse primers around F87-116 and R202-231, respectively (**Figure 2D**). F87-116 was used to screen all seven reverse primers, and R197-230 showed the best results (**Figure 2E**). Then R197-230 was used to screen all seven forward primers, and F91-121 showed the best results (**Figure 2F**). Eventually, the optimal primer pair F91-121/R197-230 was screened out.

### 3.3 Analytical specificity

**Figure 3:**
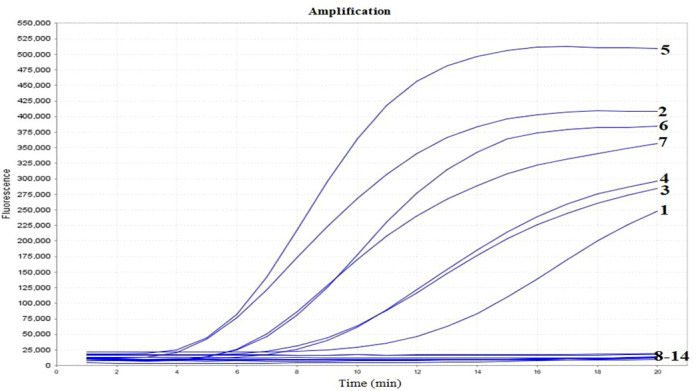
Specificity of the real-time RT-RAA assay. Curves 1-14, nucleic acid templates corresponding to H1N1, H3N2, H5N1, H5N6, H7N9, H9N2, H11N3, IBV-V, IBV-Y, ICV, RSV-A, RSV-B, SARS-CoV-2 and negative control, respectively.

Specificity analysis indicated that the real-time RT-RAA assay was positive for H1N1, H3N2, H5N1, H5N6, H7N9, H9N2, H11N3 and negative for IBV-V, IBV-Y, ICV, RSV-A, RSV-B, and SARS-CoV-2, and negative groups (**Figure 3**). These results indicate that the established real-time RT-RAA assay is specific to IAVs.

### 3.4 Analytical sensitivity

**Figure 4:**
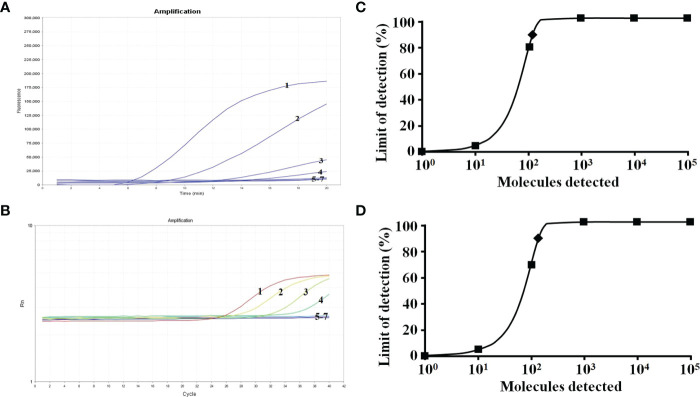
Sensitivity tests for IAVs. Curves 1–7, correspond to 10^5^–10^0^ copies and the negative control, respectively. **(A)** Results of the real-time RT-RAA assay. **(B)** Results of the RT-qPCR assay. **(C)** The detection limit of the real-time RT-RAA assay at 95% reliability (142 copies per reaction) is labeled with a rhomboid. **(D)** The detection limit of the RT-qPCR assay at 95% reliability (161 copies per reaction) is labeled with a rhomboid.

Sensitivity analysis of the real-time RT-RAA assay using serial dilutions of pMD18-T-M plasmid (10^5^–10^0^ copies per reaction) as templates. For comparison, the same template was tested in parallel using the RT-qPCR assay. The detection limit for both assays was 100 copies per reaction (**Figures 4A, B**). Probit regression analyses further indicated that the detection limits of the real-time RT-RAA and RT-qPCR assays at 95% probability were 142 and 161 copies per reaction for IAVs, respectively (**Figures 4C, D**).

### 3.5 Detection of clinical samples

One hundred and twenty clinical samples (lung tissue material, cloacal swab, oropharyngeal swab) were tested by real-time RT-RAA and RT-qPCR assays to evaluate the clinical performance. As shown in **Table 2**, the sensitivity and specificity of the real-time RT-RAA assay were 100% (60/60) and 100.0% (60/60), respectively. The two assays showed a very good correlation with a Kappa value of 1 (*P* < 0.001, **Table 2**). The positive predictive value (PPV) and the negative predictive value (NPV) were both 100%.

**Table 2:** Comparison of IAVs real-time RT-RAA with RT-qPCR assay on clinical samples.

Assay		RT-qPCR		Sensitivity	Specificity	Kappa
		Positive	Negative			
Real-time RT-RAA	Positive	60	0	100%	100%	1
	Negative	0	60			
	Total (120)	60	60			

## 4 Discussion

IAVs infect a variety of avian and mammalian hosts and may cause a rare pandemic in humans (Smith et al., 2009; Ciminski et al., 2020; Chauhan and Gordon, 2022). Four influenza pandemics caused by IAVs since the 20th century have brought enormous burdens to people’s lives. The 1918 influenza pandemic was caused by H1N1, commonly known as the “Spanish flu”, which is the most severe influenza pandemic to date; the “Asian flu” in 1957 was caused by the global prevalence of the H2N2 influenza virus; the influenza pandemic caused by H3N2 in 1968, commonly known as the “Hong Kong flu”; the 2009 influenza pandemic caused by H1N1, commonly known as “Swine flu,” started in Mexico, causing severe illness in otherwise healthy adults, and quickly spread to 214 countries, overseas regions or communities (Girard et al., 2010; Krammer et al., 2018; Linster et al., 2019; Akin and Gözel, 2020). Fast and reliable detection of IAVs is critical for controlling the spread of this disease. Virus culture is a high sensitivity and high specificity method, but requires qualified personnel, is time-consuming and is commonly used in the laboratory; rapid antigen testing is simple and has high specificity, but low-to-moderate sensitivity (Uyeki et al., 2022). RT-qPCR detection is a high sensitivity and specificity method, and it plays an important role in the detection of IAVs. However, it requires complex and expensive devices, professional operators, and time consumption. These conventional methods have some disadvantages, and cannot meet the needs for early and rapid clinical diagnosis. Therefore, it is necessary to establish a faster, simple, and more reliable diagnostic method to detect IAVs.

Previous studies have established RT-RAA detection methods for H5 and H7 subtypes of avian influenza viruses (Wang et al., 2020; Wang et al., 2022), and providing a rapid, sensitive and reliable detection tool for H5 and H7 subtypes of avian influenza viruses. However, there is no universal RT-RAA detection method for all IAVs. Here, we developed a novel real-time RT-RAA assay, targeting the *M* gene (highly conserved position), for the rapid detection of IAVs. During the primer and probe design stage, the *M* gene sequences of twelve representative IAVs strains were aligned. We first picked an ideal exo probe (p122-170), in which the two T residues within p122-170 labeled with a fluorophore (FAM) and quencher (BHQ1) matched 100% with all twelve representative IAVs strains. Then, RAA primers were designed according to the following basic principles: the primer length should be greater than or equal to 30 bp, preferably between 30 and 38 bp; the length of the amplicon should not exceed 500 bp, preferably between 100 and 200 bp; the GC content should be greater than 30%, less than 70%, preferably between 40% and 60%; the last base at the 3’ end of the primer should be very conserved; it is best to avoid short sequences with many repeats in the primer; avoid the primer directly forming a hairpin structure, or the formation of primer dimers. To ensure the efficiency of RAA amplification, a large number of primers need to be screened. We adopted the primer screening strategy reported in a previous study (Tu et al., 2022). Briefly, we used a forward primer (we randomly selected) to screen all the reverse primers, and the best reverse primer was picked out, and then used to pick all the forward primers. After secondary primer screening, the optimal primer pair F91-121/R197-230 was screened. The real-time RT-RAA assay exhibited a detection limit of 142 copies of recombinant plasmid per reaction at 95% probability, which is similar to that of the RT-qPCR assay developed by (detection limit of 161 copies of recombinant plasmid per reaction at 95% probability) (Yang et al., 2022). To evaluate the clinical performance, one hundred and twenty clinical samples (lung tissue material, cloacal swab, oropharyngeal swab) were detected by real-time RT-RAA and RT-qPCR assays, 60 samples were confirmed to be IAVs positive (CT value, ranging from 12 to 37) including 18 weak positives (CT value > 35). 60 samples were confirmed to be IAVs negative (CT value, undetermined). The result showed that the overall coincidence between the two methods was 100%.

In conclusion, a real-time RT-RAA assay, targeting conserved positions in the *M* gene of IAVs, was successfully established to detect IAVs. This assay can detect IAVs rapidly, simply and reliably, which can provide a powerful and valuable tool for the detection of IAVs, especially in resource-limited settings and POCT.

## Data availability statement

The original contributions presented in the study are included in the article/**Supplementary Material.** Further inquiries can be directed to the corresponding authors.

## Author contributions

ZG, JunL and JL designed the project. HC, CZ, KZ, JP, YK and FT performed the experiments. Data were analyzed by ZC, YS, YW, CL and LZ. Manuscript drafted by CZ and FT. ZG, HC revised the manuscript. All authors contributed to the article and approved the submitted version.
