# Supplementary material for: Rapid detection of influenza A viruses using a real-time reverse transcription recombinase-aided amplification assay
Source: Front Cell Infect Microbiol. 2023 Jan 5;12:1071288. doi: 10.3389/fcimb.2022.1071288 (PMC9849684; doi:10.3389/fcimb.2022.1071288)
Supplement: Supplementary file 1 [file DataSheet_1.docx]

**Supplementary Table 1.** Details of clinical samples

| **Serial number** | **Source** | **Time** | **Host** | **Location** |
| --- | --- | --- | --- | --- |
| 1 | Lung tissue material | 2021..01.25 | Chicken | China: Hebei |
| 2 | Lung tissue material | 2021..01.25 | Chicken | China: Hebei |
| 3 | Lung tissue material | 2021..01.25 | Chicken | China: Hebei |
| 4 | Lung tissue material | 2021..01.25 | Chicken | China: Hebei |
| 5 | Lung tissue material | 2021..01.25 | Chicken | China: Hebei |
| 6 | Cloacal swab | 2021..01.25 | Chicken | China: Hebei |
| 7 | Cloacal swab | 2021..01.25 | Chicken | China: Hebei |
| 8 | Oropharyngeal swab | 2021..01.25 | Chicken | China: Hebei |
| 9 | Oropharyngeal swab | 2021..01.25 | Chicken | China: Hebei |
| 10 | Lung tissue material | 2021.01.29 | Goose | China: Hebei |
| 11 | Lung tissue material | 2021.01.29 | Goose | China: Hebei |
| 12 | Lung tissue material | 2021.01.29 | Goose | China: Hebei |
| 13 | Lung tissue material | 2021.01.29 | Goose | China: Hebei |
| 14 | Lung tissue material | 2021.01.29 | Goose | China: Hebei |
| 15 | Lung tissue material | 2021.01.29 | Goose | China: Hebei |
| 16 | Lung tissue material | 2021.01.29 | Goose | China: Hebei |
| 17 | Oropharyngeal swab | 2021.01.29 | Goose | China: Hebei |
| 18 | Oropharyngeal swab | 2021.02.06 | Chicken | China: Hebei |
| 19 | Cloacal swab | 2021.02.06 | Chicken | China: Hebei |
| 20 | Lung tissue material | 2021.02.06 | Chicken | China: Hebei |
| 21 | Lung tissue material | 2021.02.06 | Chicken | China: Hebei |
| 22 | Lung tissue material | 2021.02.06 | Chicken | China: Hebei |
| 23 | Lung tissue material | 2021.02.06 | Chicken | China: Hebei |
| 24 | Lung tissue material | 2021.02.06 | Chicken | China: Hebei |
| 25 | Lung tissue material | 2021.02.06 | Chicken | China: Hebei |
| 26 | Cloacal swab | 2021.02.06 | Chicken | China: Hebei |
| 27 | Cloacal swab | 2021.02.06 | Chicken | China: Hebei |
| 28 | Cloacal swab | 2021.02.06 | Chicken | China: Hebei |
| 29 | Lung tissue material | 2021.02.06 | Chicken | China: Hebei |
| 30 | Oropharyngeal swab | 2021.03.02 | Duck | China: Hebei |
| 31 | Oropharyngeal swab | 2021.03.02 | Duck | China: Hebei |
| 32 | Oropharyngeal swab | 2021.03.02 | Duck | China: Hebei |
| 33 | Oropharyngeal swab | 2021.03.02 | Duck | China: Hebei |
| 34 | Oropharyngeal swab | 2021.03.02 | Duck | China: Hebei |
| 35 | Oropharyngeal swab | 2021.03.02 | Duck | China: Hebei |
| 36 | Oropharyngeal swab | 2021.03.02 | Duck | China: Hebei |
| 37 | Oropharyngeal swab | 2021.03.02 | Duck | China: Hebei |
| 38 | Lung tissue material | 2021.03.02 | Duck | China: Hebei |
| 39 | Lung tissue material | 2021.03.02 | Duck | China: Hebei |
| 40 | Lung tissue material | 2021.03.02 | Duck | China: Hebei |
| 41 | Lung tissue material | 2021.03.02 | Duck | China: Hebei |
| 42 | Lung tissue material | 2021.03.02 | Duck | China: Hebei |
| 43 | Lung tissue material | 2021.03.02 | Duck | China: Hebei |
| 44 | Oropharyngeal swab | 2021.03.02 | Duck | China: Hebei |
| 45 | Oropharyngeal swab | 2021.03.02 | Duck | China: Hebei |
| 46 | Lung tissue material | 2021.11.25 | Chicken | China: Hebei |
| 47 | Lung tissue material | 2021.11.25 | Chicken | China: Hebei |
| 48 | Lung tissue material | 2021.11.25 | Chicken | China: Hebei |
| 49 | Lung tissue material | 2021.11.25 | Chicken | China: Hebei |
| 50 | Cloacal swab | 2021.11.25 | Chicken | China: Hebei |
| 51 | Cloacal swab | 2021.11.25 | Chicken | China: Hebei |
| 52 | Cloacal swab | 2021.11.25 | Chicken | China: Hebei |
| 53 | Cloacal swab | 2021.11.25 | Chicken | China: Hebei |
| 54 | Cloacal swab | 2021.11.25 | Chicken | China: Hebei |
| 55 | Lung tissue material | 2021.11.26 | Goose | China: Hebei |
| 56 | Lung tissue material | 2021.11.26 | Goose | China: Hebei |
| 57 | Lung tissue material | 2021.11.26 | Goose | China: Hebei |
| 58 | Lung tissue material | 2021.11.26 | Goose | China: Hebei |
| 59 | Lung tissue material | 2021.11.26 | Goose | China: Hebei |
| 60 | Lung tissue material | 2021.12.09 | Chicken | China: Hebei |
| 61 | Lung tissue material | 2021.12.09 | Chicken | China: Hebei |
| 62 | Lung tissue material | 2021.12.09 | Chicken | China: Hebei |
| 63 | Lung tissue material | 2021.12.09 | Chicken | China: Hebei |
| 64 | Oropharyngeal swab | 2021.12.09 | Chicken | China: Hebei |
| 65 | Oropharyngeal swab | 2021.12.09 | Chicken | China: Hebei |
| 66 | Oropharyngeal swab | 2021.12.09 | Chicken | China: Hebei |
| 67 | Oropharyngeal swab | 2021.12.09 | Chicken | China: Hebei |
| 68 | Lung tissue material | 2021.12.09 | Chicken | China: Hebei |
| 69 | Lung tissue material | 2021.12.09 | Chicken | China: Hebei |
| 70 | Lung tissue material | 2021.12.09 | Chicken | China: Hebei |
| 71 | Lung tissue material | 2021.12.09 | Chicken | China: Hebei |
| 72 | Cloacal swab | 2021.12.09 | Goose | China: Hebei |
| 73 | Cloacal swab | 2021.12.28 | Goose | China: Hebei |
| 74 | Lung tissue material | 2021.12.28 | Goose | China: Hebei |
| 75 | Lung tissue material | 2021.12.28 | Goose | China: Hebei |
| 76 | Lung tissue material | 2021.12.28 | Goose | China: Hebei |
| 77 | Cloacal swab | 2021.12.28 | Goose | China: Hebei |
| 78 | Cloacal swab | 2021.12.28 | Goose | China: Hebei |
| 79 | Cloacal swab | 2021.12.28 | Goose | China: Hebei |
| 80 | Cloacal swab | 2022.02.12 | Duck | China: Hebei |
| 81 | Cloacal swab | 2022.02.12 | Duck | China: Hebei |
| 82 | Cloacal swab | 2022.02.12 | Duck | China: Hebei |
| 83 | Cloacal swab | 2022.02.12 | Duck | China: Hebei |
| 84 | Cloacal swab | 2022.02.12 | Duck | China: Hebei |
| 85 | Lung tissue material | 2022.02.12 | Duck | China: Hebei |
| 86 | Lung tissue material | 2022.02.12 | Duck | China: Hebei |
| 87 | Lung tissue material | 2022.02.12 | Duck | China: Hebei |
| 88 | Cloacal swab | 2022.02.12 | Duck | China: Hebei |
| 89 | Cloacal swab | 2022.02.12 | Duck | China: Hebei |
| 90 | Cloacal swab | 2022.02.12 | Duck | China: Hebei |
| 91 | Oropharyngeal swab | 2022.02.28 | Chicken | China: Hebei |
| 92 | Oropharyngeal swab | 2022.02.28 | Chicken | China: Hebei |
| 93 | Oropharyngeal swab | 2022.02.28 | Chicken | China: Hebei |
| 94 | Oropharyngeal swab | 2022.02.28 | Chicken | China: Hebei |
| 95 | Oropharyngeal swab | 2022.02.28 | Chicken | China: Hebei |
| 96 | Oropharyngeal swab | 2022.02.28 | Chicken | China: Hebei |
| 97 | Oropharyngeal swab | 2022.02.28 | Chicken | China: Hebei |
| 98 | Oropharyngeal swab | 2022.02.28 | Chicken | China: Hebei |
| 99 | Oropharyngeal swab | 2022.02.28 | Chicken | China: Hebei |
| 100 | Oropharyngeal swab | 2022.02.28 | Chicken | China: Hebei |
| 101 | Lung tissue material | 2021.11.26 | Chicken | China: Jilin |
| 102 | Lung tissue material | 2021.11.26 | Chicken | China: Jilin |
| 103 | Lung tissue material | 2021.11.26 | Chicken | China: Jilin |
| 104 | Oropharyngeal swab | 2021.11.26 | Chicken | China: Jilin |
| 105 | Oropharyngeal swab | 2021.11.26 | Chicken | China: Jilin |
| 106 | Oropharyngeal swab | 2021.12.17 | Chicken | China: Jilin |
| 107 | Lung tissue material | 2021.12.17 | Chicken | China: Jilin |
| 108 | Lung tissue material | 2021.12.17 | Chicken | China: Jilin |
| 109 | Lung tissue material | 2021.12.17 | Chicken | China: Jilin |
| 110 | Lung tissue material | 2021.12.17 | Chicken | China: Jilin |
| 111 | Lung tissue material | 2022.01.26 | Goose | China: Jilin |
| 112 | Lung tissue material | 2022.01.26 | Goose | China: Jilin |
| 113 | Lung tissue material | 2022.01.26 | Goose | China: Jilin |
| 114 | Lung tissue material | 2022.01.26 | Goose | China: Jilin |
| 115 | Oropharyngeal swab | 2022.01.26 | Goose | China: Jilin |
| 116 | Oropharyngeal swab | 2022.01.26 | Goose | China: Jilin |
| 117 | Oropharyngeal swab | 2022.01.26 | Goose | China: Jilin |
| 118 | Lung tissue material | 2022.03.27 | Chicken | China: Jilin |
| 119 | Lung tissue material | 2022.03.27 | Chicken | China: Jilin |
| 120 | Lung tissue material | 2022.03.27 | Chicken | China: Jilin |
